# Supplementary material for: Antimicrobial stewardship interventions in least developed and low-income countries: a systematic review protocol
Source: BMJ Open. 2021 Aug 12;11(8):e047312. doi: 10.1136/bmjopen-2020-047312 (PMC8362704; doi:10.1136/bmjopen-2020-047312)
Supplement: Author's manuscript [file bmjopen-2020-047312.draft_revisions.pdf]

BMJ Open is committed to open peer review. As part of this commitment we make the peer review history of every article we publish publicly available.

When an article is published we post the peer reviewers' comments and the authors' responses online. We also post the versions of the paper that were used during peer review. These are the versions that the peer review comments apply to.

The versions of the paper that follow are the versions that were submitted during the peer review process. They are not the versions of record or the final published versions. They should not be cited or distributed as the published version of this manuscript.

BMJ Open is an open access journal and the full, final, typeset and author-corrected version of record of the manuscript is available on our site with no access controls, subscription charges or pay-per-view fees (http://bmjopen.bmj.com).

If you have any questions on BMJ Open's open peer review process please email info.bmjopen@bmj.com

BMJ Open

Antimicrobial Stewardship Interventions in Least Developed and Low-Income Countries: A Systematic Review Protocol

Journal:	*BMJ Open*
Manuscript ID	bmjopen-2020-047312
Article Type:	Protocol
Date Submitted by the Author:	25-Nov-2020
Complete List of Authors:	Mzumara, Grace; Malawi-Liverpool-Wellcome Trust Clinical Research Programme, Child Health ; University of Malawi College of Medicine, Peadiatrics and Child Health<br>Mambiya, Michael; Malawi-Liverpool-Wellcome Trust Clinical Research Programme, Child Health<br>Iroh Tam, Pui-Ying ; Liverpool School of Tropical Medicine, ; Malawi-Liverpool Wellcome Trust,
Keywords:	INFECTIOUS DISEASES, MICROBIOLOGY, Microbiology < NATURAL SCIENCE DISCIPLINES

SCHOLARONE™
Manuscripts

I, the Submitting Author has the right to grant and does grant on behalf of all authors of the Work (as defined in the below author licence), an exclusive licence and/or a non-exclusive licence for contributions from authors who are: i) UK Crown employees; ii) where BMJ has agreed a CC-BY licence shall apply, and/or iii) in accordance with the terms applicable for US Federal Government officers or employees acting as part of their official duties; on a worldwide, perpetual, irrevocable, royalty-free basis to BMJ Publishing Group Ltd ("BMJ") its licensees and where the relevant Journal is co-owned by BMJ to the co-owners of the Journal, to publish the Work in this journal and any other BMJ products and to exploit all rights, as set out in our licence.

The Submitting Author accepts and understands that any supply made under these terms is made by BMJ to the Submitting Author unless you are acting as an employee on behalf of your employer or a postgraduate student of an affiliated institution which is paying any applicable article publishing charge ("APC") for Open Access articles. Where the Submitting Author wishes to make the Work available on an Open Access basis (and intends to pay the relevant APC), the terms of reuse of such Open Access shall be governed by a Creative Commons licence – details of these licences and which Creative Commons licence will apply to this Work are set out in our licence referred to above.

Other than as permitted in any relevant BMJ Author's Self Archiving Policies, I confirm this Work has not been accepted for publication elsewhere, is not being considered for publication elsewhere and does not duplicate material already published. I confirm all authors consent to publication of this Work and authorise the granting of this licence.

For peer review only

Antimicrobial Stewardship Interventions in Least Developed and Low-Income Countries: A Systematic review Protocol

Author Information
First Author: Grace Mzumara, gmzumara@mlw.mw

Institutional Affiliation: University of Malawi College of Medicine, Malawi Liverpool Wellcome Trust Clinical Research Programme

Authors

1. Michael Mambiya, mmambiya@mlw.mw

Institutional Affiliation: Malawi Liverpool Wellcome Trust Clinical Research programme.

2. Pui-Ying Iroh Tam, irohtam@mlw.mw

Affiliation: Malawi-Liverpool Wellcome Trust Clinical Research Programme, [2]University of Malawi, College of Medicine, [3]Liverpool School of Tropical Medicine

Corresponding Author: Grace Mzumara

Corresponding Authors email and address:

gmzumara@mlw.mw

Malawi-Liverpool Wellcome Trust Clinical Research Programme, Queen Elizabeth Central Hospital, College of Medicine, P.O. Box 30096. Chichiri, Blantyre 3, Malawi, C. Africa

Abstract
Introduction

Antimicrobial resistance (AMR) is increasing in low resource settings. It complicates the management of infectious diseases and is an increasing cause of death. This is due to, among other things, lack of health resources for appropriate diagnosis and unregulated access to antimicrobials in the public sphere. Developing context-specific interventions that enable judicious use of antimicrobials is important to curb this problem.

Methods

We will conduct a narrative review of AMS approaches in Development Assistance Committee least developed and low-income countries. We will include AMS interventions aimed at hospitalised patients in all age groups. We will exclude community-based trials and studies that solely focus on viral, fungal or parasite infections. All AMS interventions will be classified as structural, enabling, persuasive, restrictive, or combined. Outcomes of included studies will be classified as clinical, microbiological, or behavioural outcomes. The studies to be included will be randomised controlled trials, controlled before and After trials, interrupted time series trials, cohort, and qualitative studies. Data will be extracted using forms adapted from the Cochrane collaboration data collection form. This systematic review will be conducted according to the Preferred Reporting Items for Systematic Reviews and Meta-Analyses (PRISMA) guidelines

For peer review only

and risk of bias will be done according to the Integrated quality Criteria for Review of Multiple Study Designs (ICROMS).

Ethics and Dissemination

Our findings will be presented to clinicians and policy makers, to support developing AMS protocols for low resource settings. We will publish our results in peer reviewed journals.

Prospero Registration Number: CRD42020210634

Key words: antimicrobial stewardship, antimicrobial resistance, low income countries, low resource setting.

Article Summary

Strengths and Limitations of this study

- This study will identify Antimicrobial Stewardship Practices in low income and least developed countries.
- We will describe the outcomes and impact of identified AMS practices.
- Risk of bias will be assessed using the Integrated quality Criteria for Review of Multiple Study Designs
- Expected limitations include the variation in outcome measures, differences in the quality of the studies identified and publication bias.

Introduction

Infectious diseases are among the top leading causes of death worldwide and predominantly affect children in low and middle income countries (1–5). A major challenge to managing infectious diseases in these settings is the growing resistance to current antimicrobials (6).

A large surveillance study in Malawi, found that antibacterial resistance to first line antibiotics (amoxycillin, chloramphenicol and cotrimoxazole) increased between 1998 and 2016 (7). About 40% of all isolates were resistant to one or two first line antibiotics (7). Among children aged less than 5 years, resistance to first line antibiotics from gram negative organisms rose from 3.4% to 30.2 %, and from 5.9% to 93.7% for Klebsiella species (6). Drug resistant gram negative bacteria are particularly concerning as they are associated with increasing mortality in low income countries (5,8,9).

Antimicrobial resistance complicates the management of common infectious diseases in these settings. For example, the majority of cases and deaths from bacterial meningitis occur in children from less developed countries (3). However antibiotic resistance (AMR) among antimicrobials used to treat *Streptococcus pneumoniae*, *Haemophilus Influenza* or *Neisseria Meningitidis* is found at presumed higher rates in low resource countries (3,10). Other examples are diarrhoeal diseases that are the are leading causes of death and disability in developing countries where enteric pathogens are found to be resistant to commonly prescribed antimicrobials (1,11,12). Among neonates the rising exposure to prophylactic antibiotics in pregnant women and very low birth weight infants raises concerns about possible antibiotic resistant neonatal sepsis which could increase cases and deaths from Group B Streptococcus (2,8,13–15).

For peer review only

Several factors contribute to AMR in developing countries. Firstly, the lack of financial resources and expertise for blood culture and antimicrobial sensitivity analyses is a challenge to appropriate prescription and surveillance (8,10,11,13). Secondly, the use of prescription antibiotics is often unregulated and antibiotics can be obtained without prescriptions in many developing countries (8,13,16). In these settings, cheaper alternatives are used in place of effective antibiotics and results in AMR in hospitals and communities (13). This can cause changes in the pathogen profile of a population and perpetuate AMR (8,13). Third is the lack of appropriate policies to regulate prescribing practices which means that efforts to mitigate the problem are uncoordinated and ineffective (8,16). Efforts to mitigate these challenges are important to prevent cases and deaths from infections attributable to AMR.

These efforts are Anti-Microbial Stewardship (AMS) programs which are coordinated interventions aimed at promoting appropriate prescription of antibiotics (17,18). Structural interventions involve new diagnostic tests, persuasive interventions target behavioural change in prescribing practices, enabling interventions educate prescribers and restrictive interventions involve regulations for the usage of some antimicrobials (17,18). AMS strategies can use these classes of interventions individually or in combination (17,18). Other measures have incorporated clean water and hygiene practices for infection prevention (8,13). All AMS strategies use guidelines with defined recommendations measured as quality indicators for each intervention (16,18,19).

The World Health Organisation recommends surveillance systems for AMR to detect bacterial infections, identify resistance patterns and monitor interventions (20). To do this, context specific policies or protocols should be developed in order to guide acceptable and effective AMS solutions (16–18,21). Although studies of different AMS practices have shown that they work to improve prescribing practices there is little evidence to guide AMS in low resource settings (4,17,18).

A review of AMS interventions in low and middle income countries showed that most studies on AMS interventions report a positive effect for hospitalised patients (17). However only two low income countries were represented in this review and no recommendations could be made for AMS interventions because of the variation of strategies reviewed (17). Another review that studied the scope of AMS in children did not specifically identify interventions in low resource settings (22). There is a paucity of data to guide AMS interventions in low income countries although they face the most risk of infectious disease and AMR (4).

To address this, our systematic review aims to identify protocols, policies, or procedures for antimicrobial stewardship in hospital settings in least developed countries, defined by the Development Assistance Criteria (DAC) (23). The aim of this systematic review is to answer the following research question: What are the protocols, policies, or procedures for antimicrobial stewardship for hospitalised patients of all ages in least developed and low-income countries? Secondly, we aim to describe the impact of antimicrobial stewardship interventions on prescribing practices, clinical outcomes and antimicrobial resistance in least developed and low-income countries.

For peer review only

Methods

Protocol and Registration

The systematic review protocol was registered PROSPERO, the International Prospective Register of Systematic Reviews with the number: CRD42020210634. It will be conducted according to Preferred Reporting Items for Systematic Reviews and Meta-analyses checklist (24). Amendments that may be made at any stage of the study will be reflected in amendments to the register.

Study Design

We will conduct a narrative review of AMS approaches in DAC least and low-income settings. We expect significant variation in the types of interventions studied and, in the methods, and populations studied. Therefore, given anticipated limited data, there may not be sufficient studies to conduct a meta-analysis of interventions and outcomes.

Eligibility Criteria

Population

The Population under study are neonates, children and adults in least developed and low income countries as classified by Development Assistance Committee (DAC) Criteria (23). We will include studies about hospital based AMS approaches including surgical patients. Trials on long term patients and community-based patients will be excluded (Table 1).

Intervention

The intervention of interest is all AMS Stewardship approaches, policies, practices or regulations in place to ensure judicious prescription of antimicrobials (Table 1). We will include studies for which the intervention is clearly applied to the specified population. Reviews of interventions will not be included.

Interventions will be described as structural, enabling, persuasive, restrictive or combined according to the Cochrane Interventions to improve antibiotic prescribing practices for hospital inpatients (25). Structural interventions include using digital records, rapid antimicrobial diagnostics, or inflammatory markers to guide antimicrobial prescribing. Persuasive interventions include audits of prescription practices of health care workers and feedbacking information on where they can improve. Enabling interventions involve actively educating health care workers through treatment guideline, training modules and reminders (posters or cards). Restrictive interventions involve authorising antimicrobial prescriptions, necessitating antibacterial sensitivity analyses or automatically discontinuing antimicrobial therapy for certain conditions after a specified period. Combined or bundled interventions will be those that combine at least two types of these interventions.

All interventions involving hygiene and sanitation will be described as infection prevention strategies. Studies with information on infection prevention will be included if they are incorporated as part of at least one type AMS intervention described above.

Comparator

There is no comparator for this systematic review.

Outcome

The outcomes of interest will be categorised into behavioural, clinical, and microbiological outcomes (Table 1). Behavioural outcomes include changes in prescribing practices among

For peer review only

medical personnel and compliance to AMS policies or protocols. Where available, a metric will be used to describe changes in prescribing practices e.g. daily doses per 100 bed-days.

Clinical outcomes include the following: Treatment success will be defined as the successful eradication of a specified pathogen from an individual by microbiological confirmation or by clinical improvement described by a study physician. Clinical outcomes will also include mortality or length of stay in hospital or duration of antimicrobial use.

Microbiological outcomes include isolation of bacteria resistant to at least one antibiotic. It is expected that clinical suspicion of bacterial infection is a major indication for antibiotic prescription in the population of interest. Therefore, we will describe bacterial infection as clinical suspicion or confirmed microbiological infection. Confirmed microbiological infection will apply to studies which performed culture and, or susceptibility testing. In this way, we intend to identify AMS protocols which were applied for treatment of confirmed and suspected infections. Infection will apply to patients who had a bacterial infection as their primary illness or as a comorbidity of an underlying condition.

For peer review only

Table 1: Systematic Review Inclusion and Exclusion Criteria

PICO	Inclusion Criteria	Exclusion Criteria
**Population**	Studies on AMS studies, protocols, or policies in DAC least developed and low-income countries<br>Hospitalized patients including surgical.<br>All age groups in the population area.<br>Studied on prescribers for hospitalized patients in DAC least developed countries	Trials on patients in the communities with no history of hospital admission during the period of the trial.<br>Patients classified by respective study to be in long term care
**Intervention**	Antimicrobial stewardship approaches, programs, policies where AMS is the sole intervention or at least one element of an intervention; and/or<br>interventions targeting hospital hygiene or infection transmission control strategies.	Studies on viral, fungal or parasite infections where bacterial infection data cannot be extracted.
**Comparison**	None	None
**Outcomes**	Behavioral outcomes: changes in prescribing practices	Studies comparing the therapeutic effectiveness of one type of antimicrobial against another type. These are studies with the primary

	Microbiological outcomes: Bacterial infection confirmed by culture and sensitivity or by clinical suspicion. Confirmed microbiological infection with resistant bacteria at admission, during hospital stay, or post discharge.<br><br>Clinical outcomes: treatment success (confirmed microbiological clearance or clinical improvement) length of stay in hospital or death.	goal of determining superiority of one drug.

For peer review only

Information Sources

The data sources include PubMed, Ovid Medline, Ovid EMBASE, World Health Organisation Library Database (WHOLIS), African Index Medicus (AIM) and Cochrane Central Register of Controlled Trials (CENTRAL). We will study bibliographies in studies found to create a comprehensive list of relevant studies that might have been missed during the initial database search. Where necessary, we will contact authors for supporting information.

Types of Studies

We will include randomised-controlled trials (RCT), before-after (CBA, controlled and non-controlled) interrupted time-series (ITS, controlled and non-controlled), and cohort studies. We will also include qualitative studies that describe interventions targeting behavioural change and other relevant AMS strategies.

Search Strategy

Two independent reviewers will conduct literature search using the pre-specified terms.

Population: Low income country or Least developed country and hospital or primary care or community hospital or district hospital or tertiary hospital or tertiary care and adults or children or neonates or obstetrics or Women.

Intervention: Antimicrobial stewardship or Antimicrobial stewardship protocols or rapid antimicrobial diagnostics or antimicrobial training.

Outcome: prescription practices or compliance or treatment success or mortality or length of hospital stay or antimicrobial resistance or first line antibiotics or second line antibiotics or multidrug resistant bacterial infection.

During the review of titles and abstracts, reviewers will include publications on Antimicrobial resistance and antimicrobial stewardship in the countries listed in the DAC criteria as least developed or low income. Studies will be excluded if they are about animal studies or are exclusively primarily about viral, fungal or parasite infections, with no implications data on bacterial infections that can be extracted. We will limit this systematic review to studies

published in English. We will only include articles from studies between the year 2000 and 2020. The list of articles collected by the two reviewers will be consolidated and duplicates will be removed.

During the second phase of the review full text review and analysis will be done. Studies included will need to have occurred within the population of interest and the interventions need to have be clearly applicable to specified settings.

A third reviewer will resolve discrepancies in selection and their decision will be final. Full text review will be conducted for the selected literature.

Data Extraction, Synthesis, and management

Data extraction will be done using the Cochrane Collaboration Data collection form for intervention reviews (26). This form is a comprehensive assessment of full text reviews. Qualitative data will be extracted through a form adapted from the Cochrane guidelines for extracting qualitative information (27). The reasons for exclusion at this stage will be recorded in these forms. The process of data selection leading up to data synthesis will be presented using the PRISMA flow chart (28). Due to the expected variation in methods and outcomes, a meta-analysis will not be performed.

Information will be categorised according to the demographic group, that is, neonates, children under 18 or adults. Due to the expected variation in methods and reporting practices a narrative approach will be used to synthesize and report the data. Outcomes will be categorised according to the target of the intervention. Emerging themes from the data will be presented. A data summary table will be created to demonstrate major themes and interventions from included studies.

Before beginning the systematic review, two authors will pilot the data extraction form independently for five full review papers. Discrepancies in data collection will be consolidated and the data collection form will be adapted to reflect all information required.

Assessment of Risk of Bias

This systematic review will be conducted according to the Preferred Reporting Items for Systematic Reviews and Meta-Analyses (PRISMA) guidelines (29).

The assessment of risk of bias will be done according to the Integrated quality Criteria for Review of Multiple Study Designs (ICROMS) (30). We expect that multiple study designs could be used to describe and assess AMS strategies in low resource settings. The ICROMS tool allows for a comprehensive assessment of the quality criteria for RCT, ITS, CBA, NCBA, cohort and qualitative studies (30). This allows for the inclusion of qualitative studies which may assess behavioural change in prescribing practices as is important to AMS.

This tool assesses the quality of studies on the social and contextual determinants of an intervention and outcome. ICROMS was adopted for systematic reviews and meta-analysis in infection prevention and antimicrobial prescribing (30).

Using the ICROMS tool each study will be assessed for universally acceptable standards for its respective study type. This means randomised controlled studies will be assessed according to their own study type standards and qualitative studies will also have their own criterion. Seven dimensions describe the quality criteria each study type will be assessed for. These are:

For peer review only

clear aims and justification, managing bias in sampling or between groups, managing bias in outcome measurements and blinding, managing bias in follow-up, managing bias in other study aspects, analytical rigor and managing bias in reporting/ethical considerations. Each type of study will then be assessed on its quality criteria among thirty-three criteria (30).

Using the ICROMS tool methodological robustness of a study is analysed by assessing if the study meets the pre-specified mandatory criterion its type of study. Studies that do not meet the mandatory criteria will be excluded.

The scientific validity of each study will be assessed by calculating a minimum score of criteria met within the quality dimensions. (30). Each criterion met receives two points, if it is unclear whether the criteria is met, it is given 1 point and no points are accorded for study criteria which are clearly not met. The total quality score for each study is compared to the minimum score of its study type. The minimum score for each type of study represents 60% of the criteria required to demonstrate that the study was scientifically valid and that the outcomes are reliable (30). The minimum scores range from twenty-two points for RCT, cRCT, NCITS and NCBA, eighteen points for CBA, CITS and cohort studies, and sixteen points for qualitative studies (30). Studies not meeting the minimum score will be excluded.

The adapted ICROMS template is showed in the Appendix. This template includes a summary table for the Risk of Bias analysis of all studies. We will reference the detailed ICROMS protocol (30) when deciding on the score for each criteria.

Ethics and Dissemination

We do not expect to use patient data for this review and will not require ethical clearance. We will use the results from this review to design candidate interventions for AMS practices that can be tested this low resource setting. Our AMS protocol design will involve meeting with relevant Ministry of Health and Chikwawa Clinical officials to discuss which elements of our findings can be incorporated into an AMS strategy This review will specifically inform the development of AMS practices and policies to be tested by the Paediatric Research in Antimicrobial Stewardship and Management (PRISM) network in Chikwawa district in Malawi. Malawi is a southern-African country classified as a DAC least developed country and the incidence of AMR is rising (23,31). Finding ways to promote AMS in this setting can be useful for similar settings as well. We will publish findings from this review in peer reviewed journals and present our research to local and international conferences.

Acknowledgements

This work was supported by a Wellcome Trust Programme Grant (Grant number 091909/Z/10/Z) and the MLW Programme Core Award (Grant 206545/Z/17/Z) from the Wellcome Trust.

Conflict of Interest: None Declared

Patient Consent for Publication: None Required

Author Contributions

PI is the guarantor of the review. GM and PI conceived the topic and the structure of the protocol. GM wrote the first draft of the protocol and will coordinate subsequent systematic review activities. PI edited the manuscript and MM read and provided feedback.

For peer review only

Funding

The sponsor for this project is the Liverpool School of Tropical Medicine and it was funded by the Wellcome Trust.

Disclaimer

The Funders had no role in conceiving the project and in writing the manuscript. The funders will not provide input to collecting and analysing the data or in disseminating the results.

Keywords: Antimicrobial resistance, antimicrobial stewardship, low resource settings

Word Count: 3224

References

1. IHME. Findings from the Global Burden of Disease Study 2017. Vol. Institute, Lancet. Seattle, WA; 2018.

2. Ramasethu J, Kawakita T. Antibiotic stewardship in perinatal and neonatal care. Semin Fetal Neonatal Med [Internet]. 2017;22(5):278–83. Available from: http://dx.doi.org/10.1016/j.siny.2017.07.001

3. Fuller DG, Duke T, Shann F, Curtis N, Fuller DG, Duke T, et al. Antibiotic treatment for bacterial meningitis in children in developing countries developing countries. 2013;4936.

4. Iroh Tam P-Y, Dramowski A, Labi A-K, Mujuru HA, Ogunbosi BO. Antimicrobial resistance among children in Africa: need for paediatric clinical trials. Expert Rev Anti Infect Ther [Internet]. 2020;0(0). Available from: https://doi.org/10.1080/14787210.2020.1782741

5. Nellums LB, Thompson H, Holmes A, Castro-Sánchez E, Otter JA, Norredam M, et al. Antimicrobial resistance among migrants in Europe: a systematic review and meta-analysis. Lancet Infect Dis. 2018;18(7):796–811.

6. Tam PI, Musicha P, Kawaza K, Cornick J, Denis B, Freyne B, et al. Emerging Resistance to Empiric Antimicrobial Regimens for Pediatric Bloodstream Infections in Malawi (1998 – 2017). 2019;69(1):61–8.

7. Musicha P, Cornick JE, Bar-zeev N, French N, Masesa C, Denis B, et al. Trends in antimicrobial resistance in bloodstream infection isolates at a large urban hospital in Malawi (1998 – 2016): a surveillance study. Lancet Infect Dis [Internet]. 2017;17(10):1042–52. Available from: http://dx.doi.org/10.1016/S1473-3099(17)30394-8

8. Bebell LM, Muiru AN. Antibiotic use and emerging resistance: How can resource-limited countries turn the tide? Glob Heart. 2014;9(3):347–58.

9. Gandra S, Tseng KK, Arora A, Bhowmik B, Robinson ML, Panigrahi B, et al. The Mortality Burden of Multidrug-resistant Pathogens in India : A Retrospective , Observational Study. 2019;69(4):563–70.

10. Leopold SJ, Leth F Van, Tarekegn H, Schultsz C. Antimicrobial drug resistance among clinically relevant bacterial isolates in sub-Saharan Africa : a systematic review. 2014;(May):2337–53.

For peer review only

11. Fredrick Odhiambo, Tura Galgalo, Arvelo Wences, Onesmus Maina Muchemi, Evalyne Wambui Kanyina, Juliana Chepkemoi Tonui, Samwel Amwayi WB. Antimicrobial resistance: capacity and practices among clinical laboratories in Kenya, 2013. Pan Afr Med J [Internet]. 2014;8688:1–8. Available from: http://www.panafrican-med-journal.com/content/article/19/332/full/%0A©

12. Kariuki S, Gordon MA, Feasey N, Parry CM, Trust W, Campus G, et al. Antimicrobial resistance and management of invasive salmonella disease. Vacci. 2016;33(0 3).

13. Medugu N, Iregbu K, Tam PI, Obaro S. Aetiology of neonatal sepsis in Nigeria , and relevance of Group b streptococcus : A systematic review. PLoS One [Internet]. 2018;13(7):1–16. Available from: https://doi.org/10.1371/journal.pone.0200350%0AEditor:

14. Allegranzi B, Nejad SB, Combescure C, Graafmans W, Attar H, Donaldson L, et al. Burden of endemic health-care-associated infection in developing countries: Systematic review and meta-analysis. Lancet [Internet]. 2011;377(9761):228–41. Available from: http://dx.doi.org/10.1016/S0140-6736(10)61458-4

15. Sagori, Mukhopadhyay, Shaon, Sengupta, Karen M. P. Challenges and Opportunities for Antibiotic Stewardhsip Among Preterm Infants. Physiol Behav. 2017;176(12):139–48.

16. Nguyen Van K, Do Thi Thuy N, Chandna A, Nguyen Vu T, Pham Van C, Doan Mai P, et al. Antibiotic use and resistance in emerging economies: a situation analysis for Viet Nam. BMC Public Health [Internet]. 2013;13:1158. Available from: http://www.ncbi.nlm.nih.gov/pubmed/24325208%5CnAvailable from ProQuest in http://link.worldcat.org/?rft.institution_id=129756&spage=1158&pkgName=nhshospital&PQUEST.WAYFlessID=50332&issn=1471-2458&linkclass=to_article&jKey=44782&provider=PQUEST&date=2013-0

17. Dijck C Van, Arnoldine J. Antibiotic stewardship interventions in hospitals in low-and middle- income countries : a systematic review. World Heal Organ. 2018;(December 2017):266–80.

18. Hulscher MEJL, Prins JM. Antibiotic stewardship: does it work in hospital practice? A review of the evidence base. Clin Microbiol Infect [Internet]. 2017;23(11):799–805. Available from: https://doi.org/10.1016/j.cmi.2017.07.017

19. Bosch CMA Van Den, Hulscher MEJL, Natsch S, Wille J, Prins JM, Geerlings SE. Applicability of generic quality indicators for appropriate antibiotic use in daily hospital practice : a cross-sectional point-prevalence multicenter study. Clin Microbiol Infect [Internet]. 2016;22(10):888.e1-888.e9. Available from: http://dx.doi.org/10.1016/j.cmi.2016.07.011

20. WHO. Organization World Health. Global Strategy for Containment of Antimicrobial Resistance. World Heal Organ [Internet]. 2001;WHO/CDS/CS:105. Available from: http://scholar.google.com/scholar?hl=en&btnG=Search&q=intitle:WHO+Global+Strategy+for+Containment+of+Antimicrobial+Resistance#0%0Afile:///C:/Users/Admin/AppData/Local/Mendeley Ltd./Mendeley Desktop/Downloaded/Organização Mundial de Saúde - 2001 - ORIGINAL

21. Dyar OJ, Huttner B, Schouten J, Pulcini C, Study E. What is antimicrobial stewardship ? Clin Microbiol Infect [Internet]. 2017;23(11):793–8. Available from:

For peer review only

https://doi.org/10.1016/j.cmi.2017.08.026

22. Grammatico-Guillon L, Abdurrahim L, Shea K, Astagneau P, Pelton S. Scope of Antibiotic Stewardship Programs in Pediatrics. Clin Pediatr (Phila). 2019;58(11–12):1291–301.

23. Salvador E, Helena S. DAC List of ODA Recipients Effective for reporting on 2018 , 2019 and 2020 flows. 2021;(December 2020):2020.

24. Shamseer L, Moher D, Clarke M, Ghersi D, Liberati A, Petticrew M, Shekelle P, Stewart L P-PG. PRISMA-P (Preferred Reporting Items for Systematic review and Meta-Analysis Protocols) 2015 checklist : recommended items to address in a systematic review protocol *. Br Med J. 2015;2;349(jan02 1):g7647.

25. Davey P, Ca M, Cl S, Charani E, Mcneil K, Brown E, et al. Interventions to improve antibiotic prescribing practices for hospital inpatients (Review) SUMMARY OF FINDINGS FOR THE MAIN COMPARISON. Cochrane Database Syst Rev 2017. 2017;Art. No.:(2).

26. (EPOC). CEP and O of C. Data collection form, EPOC Resources for review authors [Internet]. 2017. Available from: https://dplp.cochrane.org/data-extraction-forms

27. S. NJL. Supplementary Guidance for Inclusion of Qualitative Research in Cochrane Systematic Reviews of Interventions [Internet]. 1st ed. Noyes J, Booth A, Hannes K, Harden A, Harris J, Lewin S LC, editor. Cochrane Collaboration Qualitative Methods Group, 2011; 2011. Available from: http://cqrmg.cochrane.org/supplemental-handbook-guidance

28. Moher D, Liberati A, Tetzlaff J, Altman DG TPG (2009). Preferred Reporting Items for Systematic Reviews and Meta-Analyses: The PRISMA Statement. PLoS Med [Internet]. 2009;6(6):(e1000097). Available from: www.prisma-statement.org

29. Shamseer L, , David Moher , Mike Clarke , Davina Ghersi , Alessandro Liberati (deceased)Petticrew M, Shekelle P, Stewart LA, Group PG. Preferred Reporting Items for Systematic review and meta-analysis protocols (Prisma-P) 2015: Elaboration and explanation. BMJ [Internet]. 2015;7647(January):1–25. Available from: http://dx.doi.org/doi:10.1136/bmj.g7647

30. Zingg W, Secci F V, Edwards R, Drumright LN, Sevdalis N, Holmes AH. Innovative tools for quality assessment : integrated quality criteria for review of multiple study designs. Public Health [Internet]. 2015;133:19–37. Available from: http://dx.doi.org/10.1016/j.puhe.2015.10.012

31. Makoka MH, Miller WC, Hoffman IF, Cholera R, Gilligan PH, Kamwendo D. Bacterial infections in Lilongwe , Malawi : aetiology and antibiotic resistance. BMC Infect Dis [Internet]. 2012;12(1):67. Available from: http://www.biomedcentral.com/1471-2334/12/67

For peer review only

Reporting checklist for protocol of a systematic review.

Based on the PRISMA-P guidelines.

Instructions to authors

Complete this checklist by entering the page numbers from your manuscript where readers will find each of the items listed below.

Your article may not currently address all the items on the checklist. Please modify your text to include the missing information. If you are certain that an item does not apply, please write "n/a" and provide a short explanation.

Upload your completed checklist as an extra file when you submit to a journal.

In your methods section, say that you used the PRISMA-Preporting guidelines, and cite them as:

Moher D, Shamseer L, Clarke M, Ghersi D, Liberati A, Petticrew M, Shekelle P, Stewart LA. Preferred Reporting Items for Systematic Review and Meta-Analysis Protocols (PRISMA-P) 2015 statement. Syst Rev. 2015;4(1):1.

		Reporting Item	Page Number
**Title**			
Identification	#1a	Identify the report as a protocol of a systematic review	1
Update	#1b	If the protocol is for an update of a previous systematic review, identify as such	n/a

For peer review only

**Registration**			
	#2	If registered, provide the name of the registry (such as PROSPERO) and registration number	1
**Authors**			
Contact	#3a	Provide name, institutional affiliation, e-mail address of all protocol authors; provide physical mailing address of corresponding author	1
Contribution	#3b	Describe contributions of protocol authors and identify the guarantor of the review	8
**Amendments**			
	#4	If the protocol represents an amendment of a previously completed or published protocol, identify as such and list changes; otherwise, state plan for documenting important protocol amendments	n/a
**Support**			
Sources	#5a	Indicate sources of financial or other support for the review	8
Sponsor	#5b	Provide name for the review funder and / or sponsor	8
Role of sponsor or funder	#5c	Describe roles of funder(s), sponsor(s), and / or institution(s), if any, in developing the protocol	8
**Introduction**			

For peer review only

Rationale	#6	Describe the rationale for the review in the context of what is already known	2 - 3
Objectives	#7	Provide an explicit statement of the question(s) the review will address with reference to participants, interventions, comparators, and outcomes (PICO)	3
**Methods**			
Eligibility criteria	#8	Specify the study characteristics (such as PICO, study design, setting, time frame) and report characteristics (such as years considered, language, publication status) to be used as criteria for eligibility for the review	3 - 5
Information sources	#9	Describe all intended information sources (such as electronic databases, contact with study authors, trial registers or other grey literature sources) with planned dates of coverage	6
Search strategy	#10	Present draft of search strategy to be used for at least one electronic database, including planned limits, such that it could be repeated	6
Study records - data management	#11a	Describe the mechanism(s) that will be used to manage records and data throughout the review	6 - 7
Study records - selection process	#11b	State the process that will be used for selecting studies (such as two independent reviewers) through each phase of the review (that is, screening, eligibility and inclusion in meta-analysis)	6 - 7

For peer review only

Study records - data collection process	#11c	Describe planned method of extracting data from reports (such as piloting forms, done independently, in duplicate), any processes for obtaining and confirming data from investigators	7
Data items	#12	List and define all variables for which data will be sought (such as PICO items, funding sources), any pre-planned data assumptions and simplifications	5
Outcomes and prioritization	#13	List and define all outcomes for which data will be sought, including prioritization of main and additional outcomes, with rationale	4
Risk of bias in individual studies	#14	Describe anticipated methods for assessing risk of bias of individual studies, including whether this will be done at the outcome or study level, or both; state how this information will be used in data synthesis	7
Data synthesis	#15a	Describe criteria under which study data will be quantitatively synthesised	n/a
Data synthesis	#15b	If data are appropriate for quantitative synthesis, describe planned summary measures, methods of handling data and methods of combining data from studies, including any planned exploration of consistency (such as I2, Kendall's τ)	n/a
Data synthesis	#15c	Describe any proposed additional analyses (such as sensitivity or subgroup analyses, meta-regression)	n/a

For peer review only

Data synthesis	#15d	If quantitative synthesis is not appropriate, describe the type of summary planned	
Meta-bias(es)	#16	Specify any planned assessment of meta-bias(es) (such as publication bias across studies, selective reporting within studies)	
Confidence in cumulative evidence	#17	Describe how the strength of the body of evidence will be assessed (such as GRADE)	7

None The PRISMA-P checklist is distributed under the terms of the Creative Commons Attribution License CC-BY 4.0. This checklist can be completed online using https://www.goodreports.org/, a tool made by the EQUATOR Network in collaboration with Penelope.ai

For peer review only

BMJ Open

Antimicrobial Stewardship Interventions in Least Developed and Low-Income Countries: A Systematic Review Protocol

Journal:	*BMJ Open*
Manuscript ID	bmjopen-2020-047312.R1
Article Type:	Protocol
Date Submitted by the Author:	29-Apr-2021

Complete List of Authors:	Mzumara, Grace; Malawi-Liverpool-Wellcome Trust Clinical Research Programme, Child Health ; University of Malawi College of Medicine, Peadiatrics and Child Health<br>Mambiya, Michael; Malawi-Liverpool-Wellcome Trust Clinical Research Programme, Child Health<br>Iroh Tam, Pui-Ying ; Malawi-Liverpool-Wellcome Trust Clinical Research Programme, Child Health; University of Malawi College of Medicine, Paediatrics and Child Health

**Primary Subject Heading**:	Infectious diseases
Secondary Subject Heading:	Public health
Keywords:	INFECTIOUS DISEASES, MICROBIOLOGY, Microbiology < NATURAL SCIENCE DISCIPLINES

SCHOLARONE™
Manuscripts

I, the Submitting Author has the right to grant and does grant on behalf of all authors of the Work (as defined in the below author licence), an exclusive licence and/or a non-exclusive licence for contributions from authors who are: i) UK Crown employees; ii) where BMJ has agreed a CC-BY licence shall apply, and/or iii) in accordance with the terms applicable for US Federal Government officers or employees acting as part of their official duties; on a worldwide, perpetual, irrevocable, royalty-free basis to BMJ Publishing Group Ltd ("BMJ") its licensees and where the relevant Journal is co-owned by BMJ to the co-owners of the Journal, to publish the Work in this journal and any other BMJ products and to exploit all rights, as set out in our licence.

The Submitting Author accepts and understands that any supply made under these terms is made by BMJ to the Submitting Author unless you are acting as an employee on behalf of your employer or a postgraduate student of an affiliated institution which is paying any applicable article publishing charge ("APC") for Open Access articles. Where the Submitting Author wishes to make the Work available on an Open Access basis (and intends to pay the relevant APC), the terms of reuse of such Open Access shall be governed by a Creative Commons licence – details of these licences and which Creative Commons licence will apply to this Work are set out in our licence referred to above.

Other than as permitted in any relevant BMJ Author's Self Archiving Policies, I confirm this Work has not been accepted for publication elsewhere, is not being considered for publication elsewhere and does not duplicate material already published. I confirm all authors consent to publication of this Work and authorise the granting of this licence.

For peer review only

ANTIMICROBIAL STEWARDSHIP INTERVENTIONS IN LEAST DEVELOPED AND LOW-INCOME COUNTRIES: A SYSTEMATIC REVIEW PROTOCOL

Grace Mzumara[1,2], Michael Mambiya[2], Pui-Ying Iroh Tam[1,2]

University of Malawi College of Medicine Department of Peadiatrics[1], Malawi Liverpool Wellcome Trust Clinical Research Programme[2]

Corresponding Author

Grace Mzumara: gmzumara@mlw.mw

Malawi-Liverpool Wellcome Trust Clinical Research Programme, Queen Elizabeth Central Hospital, College of Medicine, P.O. Box 30096. Chichiri, Blantyre 3, Malawi, C. Africa

Mike Mambiya: mmambiya@mlw.mw

Pui-Ying Iroh Tam: irohtam@mlw.mw

Word Count: 2670

For peer review only

For peer review only - http://bmjopen.bmj.com/site/about/guidelines.xhtml

ABSTRACT

Introduction

Antimicrobial resistance (AMR) is increasing in low resource settings. It complicates the management of infectious diseases and is an increasing cause of death. This is due to, among other things, lack of health resources for appropriate diagnosis and unregulated access to antimicrobials in the public sphere. Developing context-specific interventions that enable judicious use of antimicrobials is important to curb this problem.

Methods

We will conduct a systematic review of AMS approaches in Development Assistance Committee least developed and low-income countries. The inclusion criteria is antimicrobial stewardship interventions in hospitalised patients of all age groups and exclusion criteria is community-based trials, and studies that solely focus on viral, fungal or parasite infections. Antimicrobial stewardship interventions will be classified as structural, enabling, persuasive, restrictive, or combined. Outcomes of included studies will be classified as clinical, microbiological, or behavioural outcomes. The studies to be included will be randomised controlled trials, controlled before-after studies, interrupted time series trials, cohort, and qualitative studies. Data will be extracted using forms adapted from the Cochrane collaboration data collection form. This systematic review will be conducted according to the Preferred Reporting Items for Systematic Reviews and Meta-Analyses (PRISMA) guidelines and risk of bias will be done according to the Integrated quality Criteria for Review of Multiple Study Designs (ICROMS).

Ethics and Dissemination

Our findings will be presented to clinicians and policy makers, to support developing AMS protocols for low resource settings. We will publish our results in peer reviewed journals.

Prospero Registration Number: CRD42020210634

Key words: antimicrobial stewardship, antimicrobial resistance, low-income countries, low resource setting.

STRENGTHS AND LIMITATIONS OF THIS STUDY

- This study will identify Antimicrobial Stewardship Practices in low income and least developed countries.
- We will describe the outcomes and impact of identified AMS practices.
- Risk of bias will be assessed using the Integrated quality Criteria for Review of Multiple Study Designs
- Expected limitations include the variation in outcome measures, differences in the quality of the studies identified and publication bias.

For peer review only - http://bmjopen.bmj.com/site/about/guidelines.xhtml

INTRODUCTION

Infectious diseases are among the top leading causes of death worldwide and predominantly affect children in low and middle income countries.(1–5) A major challenge to managing infectious diseases in these settings is the growing resistance to current antimicrobials.(6)

A large surveillance study in Malawi, found that antimicrobial resistance to first line antibiotics (amoxycillin, chloramphenicol and cotrimoxazole) increased between 1998 and 2016.(7) About 40% of all isolates were resistant to one or two first line antibiotics.(7) Among children aged less than 5 years, resistance to first line antibiotics from gram negative organisms rose from 3.4% to 30.2 %, and from 5.9% to 93.7% for Klebsiella species.(6) Drug resistant gram negative bacteria are particularly concerning as they are associated with increasing mortality in low income countries.(5,8,9)

Antimicrobial resistance complicates the management of common infectious diseases in these settings. For example, the majority of cases and deaths from bacterial meningitis occur in children from less developed countries.(3) However antimicrobial resistance (AMR) among antimicrobials used to treat *Streptococcus pneumoniae*, *Haemophilus Influenza* or *Neisseria Meningitidis* is found at presumed higher rates in low resource countries.(3,10) Other examples are diarrhoeal diseases that are the leading causes of death and disability in developing countries where enteric pathogens are found to be resistant to commonly prescribed antimicrobials.(1,11,12) Among neonates the rising exposure to prophylactic antibiotics in pregnant women and very low birth weight infants raises concerns about possible AMR resistant neonatal sepsis which could increase cases and deaths from Group B Streptococcus.(2,8,13–15)

Several factors contribute to AMR in developing countries. Firstly, the lack of financial resources and expertise for blood culture and antimicrobial sensitivity analyses is a challenge to appropriate prescription and surveillance.(8,10,11,13) Secondly, the use of prescription antibiotics is often unregulated and antibiotics can be obtained without prescriptions in many developing countries.(8,13,16) In these settings, cheaper alternatives are used in place of effective antibiotics and results in AMR in hospitals and communities.(13) This can cause changes in the pathogen profile of a population and perpetuate AMR.(8,13) Third is the lack of appropriate policies to regulate prescribing practices which means that efforts to mitigate the problem are uncoordinated and ineffective.(8,16) Efforts to mitigate these challenges are important to prevent cases and deaths from infections attributable to AMR.

These efforts are Anti-Microbial Stewardship (AMS) practices which are coordinated interventions aimed at promoting appropriate prescription of antibiotics.(17,18) Structural interventions involve new diagnostic tests, persuasive interventions target behavioural change in prescribing practices, enabling interventions educate prescribers and restrictive interventions involve regulations for the usage of some antimicrobials.(17,18) AMS strategies can use these classes of interventions individually or in combination.(17,18) Other measures have incorporated clean water and hygiene practices for infection prevention.(8,13) All AMS strategies use guidelines with defined recommendations measured as quality indicators for each intervention.(16,18,19)

The World Health Organisation recommends surveillance systems for AMR to detect bacterial infections, identify resistance patterns and monitor interventions.(20) To do this, context specific policies or protocols should be developed in order to guide acceptable and effective

For peer review only - http://bmjopen.bmj.com/site/about/guidelines.xhtml

AMS solutions.(16–18,21) Although studies of different AMS practices have shown that they work to improve prescribing practices there is little evidence to guide AMS in low resource settings.(4,17,18)

A review of AMS interventions in low and middle income countries showed that most studies on AMS interventions report a positive effect for hospitalised patients.(17) However only two low income countries were represented in this review and no recommendations could be made for AMS interventions because of the variation of strategies reviewed.(17) Another review that studied the scope of AMS in children did not specifically identify interventions in low resource settings.(22) There is a paucity of data to guide AMS interventions in low income countries although they face the most risk of infectious disease and AMR.(4)

To address this, our systematic review aims to identify protocols, policies, or procedures for AMS in hospital settings in least developed countries, defined by the Development Assistance Criteria (DAC).(23) The aim of this systematic review is to answer the following research question: What are the interventions (protocols, policies, or procedures) for antimicrobial stewardship for hospitalised patients of all ages in least developed and low-income countries? Secondly, we aim to describe the impact of antimicrobial stewardship interventions on prescribing practices, clinical outcomes and antimicrobial resistance in least developed and low-income countries.

METHODS

Protocol and Registration

The systematic review protocol was registered PROSPERO, the International Prospective Register of Systematic Reviews with the number: CRD42020210634. It will be conducted according to Preferred Reporting Items for Systematic Reviews and Meta-analyses checklist (24). Amendments that may be made at any stage of the study will be reflected in amendments to the register.

Study Design

We will review AMS interventions in DAC least and low-income settings. We expect significant variation in the types of interventions studied and, in the methods, and populations studied. Therefore, given anticipated limited data, there may not be sufficient studies to conduct a meta-analysis of interventions and outcomes.

Eligibility Criteria

Population

The Population under study are neonates, children and adults in least developed and low income countries as classified by Development Assistance Committee (DAC) Criteria.(23) We will include studies about hospital based AMS approaches including surgical patients. Trials on long term patients and community-based patients will be excluded (Table 1).

Intervention

The intervention of interest is all AMS Stewardship approaches, policies, practices or regulations in place to ensure judicious prescription of antimicrobials (Table 1). We will include studies for which the intervention is clearly applied to the specified population. Reviews of interventions will not be included.

For peer review only - http://bmjopen.bmj.com/site/about/guidelines.xhtml

Interventions will be described as structural, enabling, persuasive, restrictive or combined according to the Cochrane Interventions to improve antibiotic prescribing practices for hospital inpatients.(25) Structural interventions include using digital records, rapid antimicrobial diagnostics, or inflammatory markers to guide antimicrobial prescribing. Persuasive interventions include audits of prescription practices of health care workers and feedbacking information on where they can improve. Enabling interventions involve actively educating health care workers through treatment guideline, training modules and reminders (posters or cards). Restrictive interventions involve authorising antimicrobial prescriptions, necessitating antibacterial sensitivity analyses or automatically discontinuing antimicrobial therapy for certain conditions after a specified period. Combined or bundled interventions will be those that combine at least two types of these interventions.

All interventions involving hygiene and sanitation will be described as infection prevention strategies. Studies with information on infection prevention will be included if they are incorporated as part of at least one type AMS intervention described above.

Comparator

There is no comparator for this systematic review.

Outcome

The outcomes of interest will be categorised into behavioural, clinical, and microbiological outcomes (Table 1). Behavioural outcomes include changes in prescribing practices among medical personnel and compliance to AMS policies or protocols. Where available, a metric will be used to describe changes in prescribing practices e.g. daily doses per 100 bed-days.

Clinical outcomes include the following: Treatment success will be defined as the successful eradication of a specified pathogen from an individual by microbiological confirmation or by clinical improvement described by a study physician. Clinical outcomes will also include mortality or length of stay in hospital or duration of antimicrobial use.

Microbiological outcomes include isolation of bacteria resistant to at least one antibiotic. It is expected that clinical suspicion of bacterial infection is a major indication for antibiotic prescription in the population of interest. Therefore, we will describe bacterial infection as clinical suspicion or confirmed microbiological infection. Confirmed microbiological infection will apply to studies which performed culture and, or susceptibility testing. In this way, we intend to identify AMS protocols which were applied for treatment of confirmed and suspected infections. Infection will apply to patients who had a bacterial infection as their primary illness or as a comorbidity of an underlying condition.

For peer review only - http://bmjopen.bmj.com/site/about/guidelines.xhtml

Table 1: Systematic Review Inclusion and Exclusion Criteria

PICO	Inclusion Criteria	Exclusion Criteria
**Population**	Studies on AMS studies, protocols, or policies in DAC least developed and low-income countries<br>Hospitalized patients including surgical.<br>All age groups in the population area.<br>Studied on prescribers for hospitalized patients in DAC least developed countries	Trials on patients in the communities with no history of hospital admission during the period of the trial.<br>Patients classified by respective study to be in long term care
**Intervention**	Antimicrobial stewardship approaches, programs, policies where AMS is the sole intervention or at least one element of an intervention; and/or<br>interventions targeting hospital hygiene or infection transmission control strategies.	Studies on viral, fungal or parasite infections where bacterial infection data cannot be extracted.
**Comparison**	None	None
**Outcomes**	Behavioral outcomes: changes in prescribing practices<br><br>Microbiological outcomes: Bacterial infection confirmed by culture and sensitivity or by clinical suspicion. Confirmed microbiological infection with resistant bacteria at admission, during hospital stay, or post discharge.<br><br>Clinical outcomes: treatment success (confirmed microbiological clearance or clinical improvement) length of stay in hospital or death.	Studies comparing the therapeutic effectiveness of one type of antimicrobial against another type. These are studies with the primary goal of determining superiority of one drug.

For peer review only

Information Sources

The data sources include PubMed, Ovid Medline, Ovid EMBASE, World Health Organisation Library Database (WHOLIS), African Index Medicus (AIM) and Cochrane Central Register of Controlled Trials (CENTRAL). We will study bibliographies in studies found to create a

For peer review only - http://bmjopen.bmj.com/site/about/guidelines.xhtml

comprehensive list of relevant studies that might have been missed during the initial database search. Where necessary, we will contact authors for supporting information.

Types of Studies

We will include randomised-controlled trials (RCT), before-after (CBA, controlled and non-controlled) interrupted time-series (ITS, controlled and non-controlled), and cohort studies. We will also include qualitative studies that describe interventions targeting behavioural change and other relevant AMS strategies.

Search Strategy

Two independent reviewers will conduct literature search using the pre-specified terms.

Population: Low income country or Least developed country and hospital or primary care or community hospital or district hospital or tertiary hospital or tertiary care and adults or children or neonates or obstetrics or Women.

Intervention: Antimicrobial stewardship or Antimicrobial stewardship protocols or rapid antimicrobial diagnostics or antimicrobial training.

Outcome: prescription practices or compliance or treatment success or mortality or length of hospital stay or antimicrobial resistance or first line antibiotics or second line antibiotics or multidrug resistant bacterial infection.

During the review of titles and abstracts, reviewers will include publications on antimicrobial resistance and antimicrobial stewardship in the countries listed in the DAC criteria as least developed or low income. Studies will be excluded if they are about animal studies or are exclusively primarily about viral, fungal or parasite infections, with no implications data on bacterial infections that can be extracted. We will limit this systematic review to studies published in English. We will only include articles from studies between the year 2000 and 2020. The list of articles collected by the two reviewers will be consolidated and duplicates will be removed.

During the second phase of the review full text review and analysis will be done. Studies included will need to have occurred within the population of interest and the interventions need to have be clearly applicable to specified settings.

A third reviewer will resolve discrepancies in selection and their decision will be final. Full text review will be conducted for the selected literature.

Data Extraction, Synthesis, and management

Data extraction will be done using the Cochrane Collaboration Data collection form for intervention reviews.(26) This form is a comprehensive assessment of full text reviews. Qualitative data will be extracted through a form adapted from the Cochrane guidelines for extracting qualitative information.(27) The reasons for exclusion at this stage will be recorded in these forms. The process of data selection leading up to data synthesis will be presented using the PRISMA flow chart.(28) Due to the expected variation in methods and outcomes, a meta-analysis will not be performed.

Information will be categorised according to the demographic group, that is, neonates, children under 18 or adults. Due to the expected variation in methods and reporting practices a narrative approach will be used to synthesize and report the data. Outcomes will be categorised according

For peer review only - http://bmjopen.bmj.com/site/about/guidelines.xhtml

to the target of the intervention. Emerging themes from the data will be presented. A data summary table will be created to demonstrate major themes and interventions from included studies.

Before beginning the systematic review, two authors will pilot the data extraction form independently for five full review papers. Discrepancies in data collection will be consolidated and the data collection form will be adapted to reflect all information required.

Assessment of Risk of Bias

This systematic review will be conducted according to the Preferred Reporting Items for Systematic Reviews and Meta-Analyses (PRISMA) guidelines.(29)

The assessment of risk of bias will be done according to the Integrated quality Criteria for Review of Multiple Study Designs (ICROMS).(30) We expect that multiple study designs could be used to describe and assess AMS strategies in low resource settings. The ICROMS tool allows for a comprehensive assessment of the quality criteria for RCT, ITS, CBA, NCBA, cohort and qualitative studies.(30) This allows for the inclusion of qualitative studies which may assess behavioural change in prescribing practices as is important to AMS.

This tool assesses the quality of studies on the social and contextual determinants of an intervention and outcome. ICROMS was adopted for systematic reviews and meta-analysis in infection prevention and antimicrobial prescribing.(30)

Using the ICROMS tool each study will be assessed for universally acceptable standards for its respective study type. This means randomised controlled studies will be assessed according to their own study type standards and qualitative studies will also have their own criterion. Seven dimensions describe the quality criteria each study type will be assessed for. These are: clear aims and justification, managing bias in sampling or between groups, managing bias in outcome measurements and blinding, managing bias in follow-up, managing bias in other study aspects, analytical rigor and managing bias in reporting/ethical considerations. Each type of study will then be assessed on its quality criteria among thirty-three criteria.(30)

Using the ICROMS tool methodological robustness of a study is analysed by assessing if the study meets the pre-specified mandatory criterion its type of study. Studies that do not meet the mandatory criteria will be excluded.

The scientific validity of each study will be assessed by calculating a minimum score of criteria met within the quality dimensions.(30) Each criterion met receives two points, if it is unclear whether the criteria is met, it is given 1 point and no points are accorded for study criteria which are clearly not met. The total quality score for each study is compared to the minimum score of its study type. The minimum score for each type of study represents 60% of the criteria required to demonstrate that the study was scientifically valid and that the outcomes are reliable (30). The minimum scores range from twenty-two points for RCT, cRCT, NCITS and NCBA, eighteen points for CBA, CITS and cohort studies, and sixteen points for qualitative studies (30). Studies not meeting the minimum score will be excluded.

The adapted ICROMS template is showed in the Appendix. This template includes a summary table for the Risk of Bias analysis of all studies. We will reference the detailed ICROMS protocol (30) when deciding on the score for each criteria.

For peer review only - http://bmjopen.bmj.com/site/about/guidelines.xhtml

ETHICS AND DISSEMINATION

We do not expect to use patient data for this review and will not require ethical clearance. We will use the results from this review to design candidate interventions for AMS practices that can be tested this low resource setting. Our AMS protocol design will involve meeting with relevant Ministry of Health and Chikwawa Clinical officials to discuss which elements of our findings can be incorporated into an AMS strategy This review will specifically inform the development of AMS practices and policies to be tested by the Paediatric Research in Antimicrobial Stewardship and Management (PRISM) network in Chikwawa district in Malawi. Malawi is a southern-African country classified as a DAC least developed country and the incidence of AMR is rising.(23,31) Finding ways to promote AMS in this setting can be useful for similar settings as well. We will publish findings from this review in peer reviewed journals and present our research to local and international conferences.

ACKNOWLEDGEMENTS

This work was supported by a Wellcome Trust Programme Grant (Grant number 091909/Z/10/Z) and the MLW Programme Core Award (Grant 206545/Z/17/Z) from the Wellcome Trust.

CONFLICT OF INTEREST: None Declared

PATIENT AND PUBLIC INVOLVEMENT: No patient Involvement

PATIENT CONSENT FOR PUBLICATION: None Required

AUTHOR CONTRIBUTIONS

PI is the guarantor of the review. GM and PI conceived the topic and the structure of the protocol. GM wrote the first draft of the protocol and will coordinate subsequent systematic review activities. PI edited the manuscript and MM read and provided feedback.

FUNDING

The sponsor for this project is the Liverpool School of Tropical Medicine and it was funded by the Wellcome Trust.

DISCLAIMER

The Funders had no role in conceiving the project and in writing the manuscript. The funders will not provide input to collecting and analysing the data or in disseminating the results.

REFERENCES

1. Intsitute for Health Metrics and Evaluation. Findings from the Global Burden of Disease Study 2017.Seattle, WA; *Institute for Health Metrics and Evaluation*. 2018.

2. Ramasethu J, Kawakita T. Antibiotic stewardship in perinatal and neonatal care. *Semin Fetal Neonatal Med*. 2017;22(5):278–83. http://dx.doi.org/10.1016/j.siny.2017.07.001

3. Fuller DG, Duke T, Shann F, et al. Antibiotic treatment for bacterial meningitis in children in developing countries developing countries. *Ann Trop Paediatr* 2013;4936.https://doi.org/10.1179/027249303225007752

For peer review only - http://bmjopen.bmj.com/site/about/guidelines.xhtml

4. Iroh Tam P-Y, Dramowski A, Labi A-K, et al. Antimicrobial resistance among children in Africa: need for paediatric clinical trials. *Expert Rev Anti Infect Ther*. 2020;0(0).https://doi.org/10.1080/14787210.2020.1782741

5. Nellums LB, Thompson H, Holmes A, et al. Antimicrobial resistance among migrants in Europe: a systematic review and meta-analysis. *Lancet Infect Dis*. 2018;18(7):796–811. DOI: 10.1016/S1473-3099(18)30219-6

6. Tam PI, Musicha P, Kawaza K, et al. Emerging Resistance to Empiric Antimicrobial Regimens for Pediatric Bloodstream Infections in Malawi (1998 – 2017).Clinical Infectious Diseases 2019;69(1):61–8.https://doi.org/10.1093/cid/ciy834

7. Musicha P, Cornick JE, Bar-zeev N, et al. Trends in antimicrobial resistance in bloodstream infection isolates at a large urban hospital in Malawi (1998 – 2016): a surveillance study. *Lancet Infect Dis*. 2017;17(10):1042–52. http://dx.doi.org/10.1016/S1473-3099(17)30394-8

8. Bebell LM, Muiru AN. Antibiotic use and emerging resistance: How can resource-limited countries turn the tide? *Glob Heart.* 2014;9(3):347–58.doi: 10.1016/j.gheart.2014.08.009

9. Gandra S, Tseng KK, Arora A, et al. The Mortality Burden of Multidrug-resistant Pathogens in India : A Retrospective , Observational Study. *Clin Inf Dis.* 2019;69(4):563–70.doi: 10.1093/cid/ciy955

10. Leopold SJ, Leth F Van, Tarekegn H, et al. Antimicrobial drug resistance among clinically relevant bacterial isolates in sub-Saharan Africa : a systematic review. *J Antimicrob Chemother*.2014;(May):2337–53.doi:10.1093/jac/dku176

11. Fredrick Odhiambo, Tura Galgalo, Arvelo Wences, et al. Antimicrobial resistance: capacity and practices among clinical laboratories in Kenya, 2013. *Pan Afr Med J.* 2014;8688:1–8.doi: 10.11604/pamj.2014.19.332.5159

12. Kariuki S, Gordon MA, Feasey N, et al. Antimicrobial resistance and management of invasive salmonella disease. *Vacci*. 2016;33(0 3).doi: 10.1016/j.vaccine.2015.03.102

13. Medugu N, Iregbu K, Tam PI, et al. Aetiology of neonatal sepsis in Nigeria , and relevance of Group b streptococcus : A systematic review. *PLoS One*. 2018;13(7):1–16. doi: 10.371/journal.pone.0200350

For peer review only - http://bmjopen.bmj.com/site/about/guidelines.xhtml

14. Allegranzi B, Nejad SB, Combescure C, et al. Burden of endemic health-care-associated infection in developing countries: Systematic review and meta-analysis. *Lancet*. 2011;377(9761):228–41. http://dx.doi.org/10.1016/S0140-6736(10)61458-4

15. Sagori, Mukhopadhyay, Shaon, Sengupta, Karen M. P. Challenges and Opportunities for Antibiotic Stewardhsip Among Preterm Infants. *Arch Dis Child Fetal Neonatal Ed*. 2017;176(12):139–48. doi: 10.1136/archdischild-2018-315412

16. Nguyen Van K, Do Thi Thuy N, Chandna A, et al. Antibiotic use and resistance in emerging economies: a situation analysis for Viet Nam. *BMC Public Health*. 2013;13:1158.doi: 10.1186/1471-2458-13-1158.

17. Dijck C Van, Arnoldine J. Antibiotic stewardship interventions in hospitals in low-and middle- income countries : a systematic review. *World Health Organisation*. 2018;(December 2017):266–80.doi: 10.2471/BLT.17.203448

18. Hulscher MEJL, Prins JM. Antibiotic stewardship: does it work in hospital practice? A review of the evidence base. *Clin Microbiol Infect*. 2017;23(11):799–805. https://doi.org/10.1016/j.cmi.2017.07.017

19. Bosch CMA Van Den, Hulscher MEJL, Natsch S, et al. Applicability of generic quality indicators for appropriate antibiotic use in daily hospital practice : a cross-sectional point-prevalence multicenter study. *Clin Microbiol Infect*. 2016;22(10):888.e1-888.e9.http://dx.doi.org/10.1016/j.cmi.2016.07.011

20. World Health Organisation. Global Strategy for Containment of Antimicrobial Resistance. *World Health Organisation*. 2001;WHO/CDS/CS:105. https://www.who.int/drugresistance/WHO_Global_Strategy.htm/en/(accessed 29 Oct 2020)

21. Dyar OJ, Huttner B, Schouten J, et al. What is antimicrobial stewardship? *Clin Microbiol Infect*. 2017;23(11):793–8. https://doi.org/10.1016/j.cmi.2017.08.026.

22. Grammatico-Guillon L, Abdurrahim L, Shea K, et al. Scope of Antibiotic Stewardship Programs in Pediatrics. *Clin Pediatr* (Phila). 2019;58(11–12):1291–301.doi: 10.1177/0009922819852985

23. Development Assistance Committe List of Official Development Assistance Recipients Effective for reporting on 2018, 2019 and 2020 flows. https://www.oecd.org/dac/financing-sustainable-development/development-finance-standards/DAC_List_ODA_Recipients2018to2020_flows_En.pdf. (accsessed Sept 20 2020).

24. Shamseer L, Moher D, Clarke M, et al. PRISMA-P (Preferred Reporting Items for Systematic review and Meta-Analysis Protocols) 2015 checklist : recommended items to address in a systematic review protocol *. Br Med J. 2015;2;349(jan02 1):g7647. doi: 10.1136/bmj.g7647

25. Davey P, Marwick C, Scott C, et al. Interventions to improve antibiotic prescribing practices for hospital inpatients Summary Of Findings For The Main Comparison.

For peer review only - http://bmjopen.bmj.com/site/about/guidelines.xhtml

Cochrane Database Syst Rev. 2017. 2017;Art. No.:(2).doi: 10.1002/14651858.CD003543.pub4.

26. Effective Practice and Organisation of Care. Data collection form, EPOC Resources for review authors. 2017. https://dplp.cochrane.org/data-extraction-forms.(accessed 20 Sept 2020)

27. Noyes J & Lewin S. Chapter 5: Extracting qualitative evidence. In: Noyes J, Booth A, Hannes K, Harden A, Harris J, Lewin S, Lockwood C (editors), Supplementary Guidance for Inclusion of Qualitative Research in Cochrane Systematic Reviews of Interventions. Version 1 (updated August 2011). *Cochrane Collaboration Qualitative Methods Group,* 2011. Available from URL http://cqrmg.cochrane.org/supplemental-handbook-guidance(accessed 11 Sept 2020)

28. Moher D, Liberati A, Tetzlaff J, et al. Preferred Reporting Items for Systematic Reviews and Meta-Analyses: The PRISMA Statement. *PLoS Med.* 2009;6(6):(e1000097).doi: 10.1371/journal.pmed.1000097

29. Shamseer L, David Moher , Mike Clarke, et al. Preferred Reporting Items for Systematic review and meta-analysis protocols (Prisma-P) 2015: Elaboration and explanation. *BMJ* . 2015;7647(January):1–25.http://dx.doi.org/doi:10.1136/bmj.g7647

30. Zingg W, Castro-Sanchez E, Secci F V, et al. Innovative tools for quality assessment : integrated quality criteria for review of multiple study designs. *Public Health*. 2015;133:19–37.doi: 10.1016/j.puhe.2015.10.012

31. Makoka MH, Miller WC, Hoffman IF. Bacterial infections in Lilongwe , Malawi : aetiology and antibiotic resistance. *BMC Infect Dis*. 2012;12(1):67. http://www.biomedcentral.com/1471-2334/12/67

For peer review only

Name of Included Study (PMID)	
	1
	2
	3
	4
	5
	6
	7

	**Criteria Mandatory**
	**X**

RCT
CBA
CITS
CS
NCITS
NCBA
QUAL

Study Design
RCT
CBA
CITS
CS
NCITS
NCBA
QUAL

For peer review only

ICROMS QUALITY CRITERIA FOR A	
Dimension	Quality criteria
Clear Aims and Justification	A
	B
	C
	D
	E
Managing bias in sampling or between groups	A
	B
	C
	D
	E
	F
Managing bias in outcome measurements and blinding	A
	B
	C
	D
	E
	F
	G
Managing bias in follow-up	A
	B
	C
Managing bias in other study aspects	A
	B
	C
	D
Analytical rigour	A
	B
	C
Managing bias in reporting / Ethical Considerations	A
	B
	C
	D
	E

**Key**
Criteria to be included in quality assessment for study design
Mandatory criteria to be met quality assessment
Criteria **NOT** to be applied in quality assessment for study design

For peer review only

Randomised - Control Trial
Controlled before-after
Controlled Interrupted time series
Cohort study
Non-controlled interupted time series
Non-controlled before-after
Qualitative

ICROMS Decision Matrix	
**Mandatory Criteria**	**Minimum Score**
1A, 2A, 2B, and 3A	22
1A, 2D, 3B and 3C	18
1A, 3D and 6A	18
1A, 2E, 3G and 4C	18
NCITS 1A, 1B, 2C and 5D	22
NCITS 1A, 1B, 2C and 5D	22
1A, 1E and 2F	16

For peer review only

\PPLICATION PER STUDY DESIGN CRITERIA

Specific Criteria	RCT
Clear Statement of the aims of the area of research	Mandatory
Rationale for number of pre- and post intervention points or adequate baseline meas	x
Explanation for lack of control group	x
Appropriateness of qualitative methodology	x
Appropriate study desing	x
Sequence Generation	Mandatory
Allocation Concealment	Mandatory
Justification for sample choice	x
Intervention and control group selection designed to protect against systematic difer	x
Comparability of groups	x
Sampling and recruitment	x
Blinding	Mandatory
Baseline Management - protection against selection bias	x
Protection against contamination	x
Protection against secular changes	x
Protection against detection bias: Blinded assessment of primary outcomes	Criteria
Reliable Outcome measures	Criteria
Comparability of outcomes	x
Follow-up of subjects (protection against exclusion bias)	Criteria
Follow-up of patients or episodes of care	Criteria
Incomplete outcome data addressed	Criteria
Protection against detection bias: Intervention unlikely to affect data collection	Criteria
Protection against information bias	x
Data collection appropriate to address research aims	x
Attempts to mitigate effects of no control	x
Sufficient data points to enable reliable statistical inference	x
Shaping of intervention effect specified	x
Analysis of sufficienty rigorous / free from bias	Criteria
Free of selective outcome reporting	Criteria
Limitatins addressed	Criteria
Conclusions clear and justified	Criteria
Free of other bias	Criteria
Ethics issues addressed	Criteria

For peer review only

For peer review only

		Study Design Criteria			
**CBA**	**CITS**	**NCITS**	**NCBA**	**CS**	**QUAL**
Mandator	Mandatory	Mandatory	Mandatory	Mandatory	Mandatory
x	Criteria	Mandatory	Mandatory	x	x
x	x	Criteria	Criteria	x	x
x	x	x	x	x	Criteria
x	x	x	x	x	Mandatory
x	x	x	x	x	x
x	x	x	x	x	x
x	x	Mandatory	Mandatory	x	x
Mandatory	x	x	x	x	x
x	x	x	x	Mandatory	x
x	x	x	x	x	Mandatory
x	x	x	x	x	x
Mandatory	x	x	x	x	x
Mandatory	x	x	x	x	x
x	Mandatory	x	x	x	x
Criteria	Criteria	Criteria	Criteria	Criteria	x
Criteria	Criteria	Criteria	Criteria	Criteria	Criteria
x	x	x	x	Mandatory	x
x	x	x	x	x	x
x	x	x	x	x	x
Criteria	Criteria	Criteria	Criteria	Mandatory	Criteria
Criteria	Criteria	Criteria	Criteria	x	x
x	x	x	x	Criteria	x
x	x	x	x	x	Criteria
x	x	Mandatory	Mandatory	x	x
x	Mandatory	x	x	x	x
x	Criteria	x	x	x	x
Criteria	Criteria	Criteria	Criteria	Criteria	Criteria
Criteria	Criteria	Criteria	Criteria	Criteria	Criteria
Criteria	Criteria	Criteria	Criteria	Criteria	Criteria
Criteria	Criteria	Criteria	Criteria	Criteria	Criteria
Criteria	Criteria	Criteria	Criteria	Criteria	Criteria
Criteria	Criteria	Criteria	Criteria	Criteria	Criteria

For peer review only

For peer review only

QUALITY CRITERIA FOR IN
**Included Study Design**
1
2
3
4
5
6
7
**Quality Assessment Summary**

For peer review only

For peer review only

ILCUDED STUDIES

Specific Criteria Met	ICROMS Score (1/2/0)
A	
B	
C	
D	
E	
A	
B	
C	
D	
E	
F	
A	
B	
C	
D	
E	
F	
G	
A	
B	
C	
A	
B	
C	
D	
A	
B	
C	
A	
B	
C	
D	
E	

**Total ICROMS Score**	
**Mimimum score met (Yes/No)**	
**Mandatory Criteria Met (Yes/No)**	
**Inclusion for systematic review systhesis (Yes /No)**	

For peer review only

Reporting checklist for protocol of a systematic review.

Based on the PRISMA-P guidelines.

Instructions to authors

Complete this checklist by entering the page numbers from your manuscript where readers will find each of the items listed below.

Your article may not currently address all the items on the checklist. Please modify your text to include the missing information. If you are certain that an item does not apply, please write "n/a" and provide a short explanation.

Upload your completed checklist as an extra file when you submit to a journal.

In your methods section, say that you used the PRISMA-Preporting guidelines, and cite them as:

Moher D, Shamseer L, Clarke M, Ghersi D, Liberati A, Petticrew M, Shekelle P, Stewart LA. Preferred Reporting Items for Systematic Review and Meta-Analysis Protocols (PRISMA-P) 2015 statement. Syst Rev. 2015;4(1):1.

For peer review only

		Reporting Item	Page Number
**Title**			
Identification	#1a	Identify the report as a protocol of a systematic review	1
Update	#1b	If the protocol is for an update of a previous systematic review, identify as such	n/a

Registration

	#2	If registered, provide the name of the registry (such as PROSPERO) and registration number	2

Authors

Contact	#3a	Provide name, institutional affiliation, e-mail address of all protocol authors; provide physical mailing address of corresponding author	1
Contribution	#3b	Describe contributions of protocol authors and identify the guarantor of the review	9

Amendments

	#4	If the protocol represents an amendment of a previously completed or published protocol, identify as such and list changes; otherwise, state plan for documenting important protocol amendments	n/a

Support

Sources	#5a	Indicate sources of financial or other support for the review	9
Sponsor	#5b	Provide name for the review funder and / or sponsor	9
Role of sponsor or funder	#5c	Describe roles of funder(s), sponsor(s), and / or institution(s), if any, in developing the protocol	9

Introduction

For peer review only

Rationale	#6	Describe the rationale for the review in the context of what is already known	3 - 4
Objectives	#7	Provide an explicit statement of the question(s) the review will address with reference to participants, interventions, comparators, and outcomes (PICO)	4
**Methods**			
Eligibility criteria	#8	Specify the study characteristics (such as PICO, study design, setting, time frame) and report characteristics (such as years considered, language, publication status) to be used as criteria for eligibility for the review	4 - 8
Information sources	#9	Describe all intended information sources (such as electronic databases, contact with study authors, trial registers or other grey literature sources) with planned dates of coverage	6
Search strategy	#10	Present draft of search strategy to be used for at least one electronic database, including planned limits, such that it could be repeated	7
Study records - data management	#11a	Describe the mechanism(s) that will be used to manage records and data throughout the review	7 - 8
Study records - selection process	#11b	State the process that will be used for selecting studies (such as two independent reviewers) through each phase of the review (that is, screening, eligibility and inclusion in meta-analysis)	7

For peer review only

Study records - data collection process	#11c	Describe planned method of extracting data from reports (such as piloting forms, done independently, in duplicate), any processes for obtaining and confirming data from investigators	7
Data items	#12	List and define all variables for which data will be sought (such as PICO items, funding sources), any pre-planned data assumptions and simplifications	6
Outcomes and prioritization	#13	List and define all outcomes for which data will be sought, including prioritization of main and additional outcomes, with rationale	5
Risk of bias in individual studies	#14	Describe anticipated methods for assessing risk of bias of individual studies, including whether this will be done at the outcome or study level, or both; state how this information will be used in data synthesis	7
Data synthesis	#15a	Describe criteria under which study data will be quantitatively synthesised	n/a
Data synthesis	#15b	If data are appropriate for quantitative synthesis, describe planned summary measures, methods of handling data and methods of combining data from studies, including any planned exploration of consistency (such as I2, Kendall's τ)	n/a
Data synthesis	#15c	Describe any proposed additional analyses (such as sensitivity or subgroup analyses, meta-regression)	n/a

For peer review only

Data synthesis	#15d	If quantitative synthesis is not appropriate, describe the type of summary planned	
Meta-bias(es)	#16	Specify any planned assessment of meta-bias(es) (such as publication bias across studies, selective reporting within studies)	
Confidence in cumulative evidence	#17	Describe how the strength of the body of evidence will be assessed (such as GRADE)	8

None The PRISMA-P checklist is distributed under the terms of the Creative Commons Attribution License CC-BY 4.0. This checklist can be completed online using https://www.goodreports.org/, a tool made by the EQUATOR Network in collaboration with Penelope.ai

For peer review only
